# A Novel Intercellular Communication-Associated Gene Signature for Prognostic Prediction and Clinical Value in Patients With Lung Adenocarcinoma

**DOI:** 10.3389/fgene.2021.702424

**Published:** 2021-08-23

**Authors:** Qin-Yu Zhao, Le-Ping Liu, Lu Lu, Rong Gui, Yan-Wei Luo

**Affiliations:** ^1^Department of Blood Transfusion, The Third Xiangya Hospital of Central South University, Changsha, China; ^2^College of Engineering and Computer Science, Australian National University, Canberra, ACT, Australia

**Keywords:** lung adenocarcinoma, intercellular communication, prognosis prediction, machine learning, tumor microenvironment, single-cell RNA-sequencing

## Abstract

**Background:**

Lung cancer remains the leading cause of cancer death globally, with lung adenocarcinoma (LUAD) being its most prevalent subtype. This study aimed to identify the key intercellular communication-associated genes (ICAGs) in LUAD.

**Methods:**

Eight publicly available datasets were downloaded from the Gene Expression Omnibus (GEO) and The Cancer Genome Atlas (TCGA) databases. The prognosis-related ICAGs were identified and a risk score was developed by using survival analysis. Machine learning models were trained to predict LUAD recurrence based on the selected ICAGs and clinical information. Comprehensive analyses on ICAGs and tumor microenvironment were performed. A single-cell RNA-sequencing dataset was assessed to further elucidate aberrant changes in intercellular communication.

**Results:**

Eight ICAGs with prognostic potential were identified in the present study, and a risk score was derived accordingly. The best machine-learning model to predict relapse was developed based on clinical information and the expression levels of these eight ICAGs. This model achieved a remarkable area under receiver operator characteristic curves of 0.841. Patients were divided into high- and low-risk groups according to their risk scores. DNA replication and cell cycle were significantly enriched by the differentially expressed genes between the high- and the low-risk groups. Infiltrating immune cells, immune functions were significantly related to ICAGs expressions and risk scores. Additionally, the changes of intercellular communication were modeled by analyzing the single-cell sequencing dataset.

**Conclusion:**

The present study identified eight key ICAGs in LUAD, which could contribute to patient stratification and act as novel therapeutic targets.

## Introduction

Lung cancer is the leading cause of cancer death and approximately 1.8 million deaths worldwide in 2020 had lung cancer as the primary cause (Sung et al., 2021). Non-small-cell lung cancer (NSCLC), one of the main histological subtypes, includes approximately 85% of the lung cancer cases. Lung adenocarcinoma (LUAD) is the most common subtype of NSCLC (Molina et al., 2008). Despite recent advances in various targeted therapies, LUAD is still characterized by a low 5-year survival rate (Ahluwalia et al., 2021). Therefore, it is crucial to identify a novel gene signature for LUAD patients’ prognosis and for the exploration of novel therapeutic targets for LUAD.

Intercellular communication, defined as the information transfer between cells, is vital for cells to grow and function normally, and may provide a unique perspective for LUAD prognosis (Mittelbrunn and Sanchez-Madrid, 2012). Cells share information by direct and indirect signaling, and the related pathways can be regulated at the gene expression level (Mittelbrunn and Sanchez-Madrid, 2012; Brucher and Jamall, 2014). Direct intercellular communication involves self-to-self communication and adjacent communication with nearby cells, while indirect intercellular communication involves local communication *via* hormones over short or large distances, respectively. Communication, occluding, and anchoring junctions are the three essential components of intercellular communication (Brucher and Jamall, 2014).

Aberrant alterations of intercellular communication in the tumor microenvironment (TME) are related to the occurrence, invasion, metastasis, and drug resistance of cancers (Xu et al., 2018; Maacha et al., 2019). Increasing evidence suggests that versatile immune cells are infiltrated in the TME of LUAD and play an essential role in cancer progression and aggressiveness (Ma et al., 2020; Tan et al., 2020). Communication between tumor cells and tumor-infiltrating immune cells may significantly affect the functions of the immune system, potentially deteriorating the clinical outcomes (Parri et al., 2020). A better understanding of the intercellular communication in TME could thus shed light on the pathogenesis and prognosis of LUAD. As a result, intercellular communication plays a significant role in many pathways and has an important impact on TME of lung cancer.

Despite the significance of intercellular communication in LUAD, it is still considered an underexplored domain. A number of previous studies have reported gene signatures with prognostic potential for LUAD, including immune (Zhang et al., 2019), hypoxia (Mo et al., 2020), and ferroptosis-related genes (Gao et al., 2021). Nevertheless, limited work has been done so far to reveal and study the intercellular communication-associated genes (ICAGs). Besides, computational methods based on single-cell RNA sequencing data have demonstrated an outstanding potential in investigating the intercellular communication in high resolution (Wang Y. et al., 2019; Efremova et al., 2020). These methods mainly focus on ligand–receptor interactions, and therefore, less attention has been given to the prognostic potential of the ICAGs.

**FIGURE 1:**
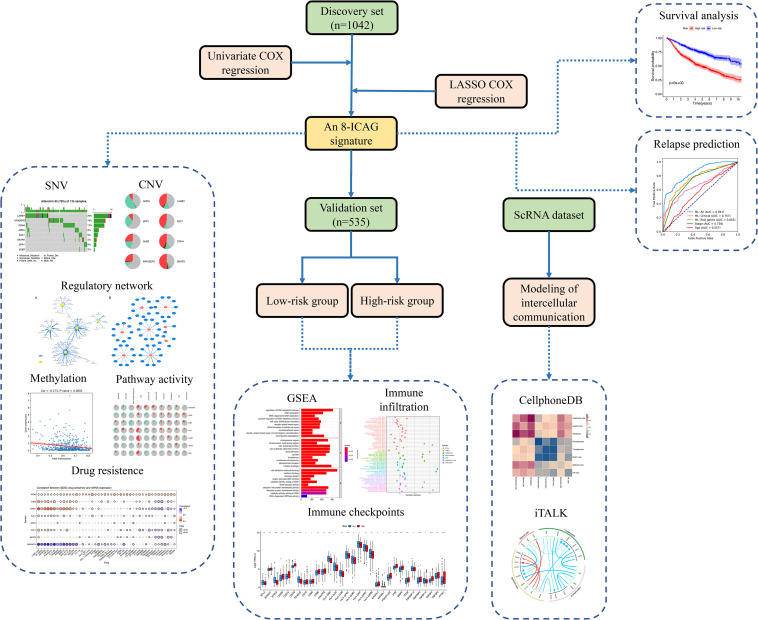
Flow chart of the design of the present study.

The present study aimed to identify the key ICAGs that could serve as prognostic markers or therapeutic targets for LUAD patients. Eight publicly available datasets were analyzed and eight LUAD prognosis ICAGs were identified. Machine learning models were then developed based on these genes and clinical information to predict the recurrence of LUAD. Comprehensive analyses on ICAGs were performed, including mutation, DNA methylation, post-transcriptional regulation, pathway activity, and drug resistance correlation analyses. Patients were divided into high- and low-risk groups according to the expression levels of these genes. Gene set enrichment analyses were performed on the differentially expressed genes (DEGs) between the high- and the low-risk groups. Tumor-infiltrating immune cells, immune functions and immune checkpoints were evaluated in different groups by using 10 different approaches. Additionally, a single-cell RNA sequencing dataset was assessed to elucidate further differences between the high- and the low-risk groups in the intercellular communication. The design of the present study was summarized in Figure 1.

## Materials and Methods

### Expression Microarray Datasets

Systematic data mining and computerized searches in the Gene Expression Omnibus (GEO) database were conducted in our study. Seven publicly available LUAD datasets, including GSE19188, GSE30219, GSE31210, GSE31546, GSE37745, GSE50081, and GSE68465, were retrieved accessing the overall survival time. The raw data were downloaded and normalized using the same methods and parameters described in the original studies. Probes with missing gene symbols were excluded. The median expression intensity was used when there were multiple probe sets mapping to the same gene symbol. Besides, gene expression data in fragments per kilobase million (FPKM) values and clinical information of the LUAD dataset from The Cancer Genome Atlas (TCGA) were also downloaded. Expression data were then transformed into the transcripts per million (TPM) values.

An empirical Bayes method was utilized to remove the batch effects by using the functions provided in the sva R package (version 3.34.0) (Leek et al., 2012). The datasets from GEO were combined and used as the discovery set, while the TCGA-LUAD cohort was used as the validation set.

### Identification of Prognosis-Related ICAGs

The gene list of ICAGs was collected based on the Kyoto Encyclopedia of Genes and Genomes (KEGG), Gene Ontology (GO), and Reactome databases (Jassal et al., 2020). A total of 440 genes were eventually downloaded from these databases. After duplicates were removed, a list of 426 ICAGs was obtained.

Univariate COX regression was used to assess ICAGs based on the discovery set, and the genes significantly correlated to the overall survival were identified with a *P*-value threshold of 0.01. The rigorous *P*-value cutoff was used to obtain a better prediction performance. Further, ICAGs with statistical significance in univariate regression were evaluated using the least absolute shrinkage and selection operator (LASSO) COX regression model. ICAGs with the best prognostic value were screened out. A risk score was then constructed according to the fitted coefficients of the LASSO COX model. The formula of the risk score was:

$$\text{RiskScore}={\text{coef}}_{1}\times {\text{ICAG}}_{1}+{\mathrm{coef}}_{2}\times {\text{ICAG}}_{2}+{\text{coef}}_{3}\times {\text{ICAG}}_{3}+\cdots$$

where the ICAG represents the normalized expression of a given ICAG, and the coef represents its coefficient in the LASSO COX model. Besides, a nomogram was also developed for convenient prediction by using the R package regplot (version 1.1).

Samples in the discovery set were divided into high- and low-risk groups according to whether their risk scores exceeded the median value. The Kaplan–Meier analysis with a log-rank test was applied to assess the prognostic difference between the two risk groups. Principal component analysis (PCA) was also performed to demonstrate and visualize the differences between the two groups. Then, the risk score and clinical variables were assessed by successively fitting the univariate and multivariate COX regression models on the GSE31210 dataset of the discovery set. It is noteworthy, that only the GSE31210 was used because it provided the most detailed clinical information. Statistical significance (*P* < 0.05) in both univariate and multivariate COX regression indicated that the risk score is an independent prognostic factor for patients with LUAD. Similarly, the validation set was also divided based on its own median value of risk scores, and the clinical significance of the risk score was also assessed on it.

### Prediction of Cancer Recurrence by Using Machine Learning

A machine-learning model called Categorical Boosting (CatBoost) was developed for the tumor recurrency prediction to further elucidate the ability of ICAGs to predict clinical outcomes. CatBoost, one of the gradient boosting algorithms, iteratively trains a weak decision tree to fit residuals of previous trees (Prokhorenkova et al., 2018). It is a powerful machine-learning technique but has yet not been widely adopted in critical care research (Zhao et al., 2020). In the present study, the ability of CatBoost to predict cancer recurrency was studied and clinical potential of ICAGs was further demonstrated. Four datasets that provided relapse information were firstly selected, including GSE68465, GSE30219, GSE50081, and GSE31210 datasets. Then, three different CatBoost models were trained based on three different feature sets. The first feature set includes only ICAGs selected by LASSO COX regression, the second one includes only clinical information such as age, gender, and stage, while the third set combined the first and the second feature sets.

Ten-fold cross-validation was performed considering the limited sample size of the utilized datasets. In particular, the dataset was randomly into 10 subsets. In each iteration, nine of them were used to train the models and the last one for validation. After 10 iterations, each subset had been validated and the validation results were then combined. The areas under receiver operator characteristic curves (AUROCs) were calculated to assess the performance of the models. Finally, the SHapley Additive exPlanation (SHAP) values were calculated according to a game theory approach to illustrate the effects of each feature on the prediction results of the third model (Lundberg et al., 2020).

### Gene Set Variant, Pathway Activity, and Regulatory Network Analyses

Gene Set Cancer Analysis (GSCA^1^) is an integrated genomic and immunogenomic online tool for gene set cancer research based on TCGA cohorts (Liu et al., 2018). The results of single-nucleotide variants (SNVs), copy number variations (CNVs), micro RNA (miRNA) network analyses, and pathway activity were obtained by uploading on the web-based platform the genes selected by LASSO COX regression and choosing the TCGA-LUAD cohort. Notably, SNV and CNV were analyzed based on the TCGA-LUAD cohort, while pathway activity and miRNA network analyses were performed on the 32 and 33 cancer types in TCGA, respectively. Additionally, the correlation of gene expression and drug sensitivity was assessed based on small molecules from the Cancer Therapeutics Response Portal (CTRP) (Seashore-Ludlow et al., 2015) and the Genomics of Drug Sensitivity in Cancer (GDSC) (Iorio et al., 2016).

The cBioPortal for Cancer Genomics^2^ provides another web-based resource for exploring, visualizing, and analyzing multidimensional cancer genomics data (Gao et al., 2013). We also used cBioPortal to explore selected ICAGs on the TCGA-LUAD cohort. Results of variant and pathway analyses were downloaded to enhance our study.

Additionally, long non-coding RNAs (lncRNAs) in the validation set were identified according to Genome Reference Consortium Human Build 38 patch release 13 (GRCh38.p13). Co-expression analysis was conducted by assessing Pearson correlation between the selected ICAGs and lncRNAs in LUAD samples. A lncRNA regulatory network was derived according to the criteria of | Correlation Coefficient| > 0.4 and *P* < 0.01 using the functions provided in the stats R package (version 3.6.0). If a ICAG have more than 10 significantly correlated lncRNAs, only 10 lncRNAs with the greatest absolute value of correlation coefficients were selected. The lncRNA network was then visualized by using the Cytoscape program.

### DNA Methylation and N6-Methyladenosine

In order to further analyze the selected ICAGs, DNA methylation data of LUAD (platform: Illumina HumanMethylation450 BeadChip) were downloaded from the TCGA database. The methylation level of CpGs was represented as β values (Bibikova et al., 2011). Pearson correlation coefficients and *P*-values were calculated between expression and methylation levels of ICAGs.

Besides, 12 N6-methyladenosine (m6A) regulatory genes were obtained *via* systematic review in published articles. The expression levels of these genes were compared by using the two-sample Wilcoxon test between the high- and the low-risk groups on the validation set.

### Gene Set Enrichment and Immunogenomic Landscape Analyses

The DEGs between the high- and the low-risk groups in the TCGA cohort, with adjusted *P*-value < 0.01 were identified using the functions provided in the stats R package (version 3.6.0). Gene set enrichment analyses based on the GO and KEGG functional and pathway terms were conducted to assess the DEGs with adjusted *P*-values threshold of 0.05, using the clusterProfiler R package (version 3.14.3) (Yu et al., 2012).

Computational methods were used to evaluate the immune infiltration and functions, including TIMER (Li et al., 2020), quanTIseq (Finotello et al., 2019), xCell (Aran et al., 2017), MCP-counter (Becht et al., 2016), EPIC (Racle et al., 2017), CIBERSORT (Newman et al., 2015) CIBERSORTx (Newman et al., 2019), and single-sample gene set enrichment analysis (ssGSEA) in an attempt to comprehensively analyze the immune differences between the two groups (Rooney et al., 2015). Additionally, a list of 79 immune checkpoint genes was obtained from Hu’s study (Hu et al., 2020), with most of these genes being ligands, receptors, or important molecules in the immune checkpoint pathways. The expression of these genes was compared between the high- and the low-risk groups by using the two-sample Wilcoxon test.

### Modeling the Intercellular Communication Based on a Single Cell RNA Sequencing Dataset

The GSE131907 dataset, which is a LUAD single-cell RNA sequencing dataset, was downloaded from the GEO database. Raw data are not available due to patient privacy concerns, and therefore, data normalized (log_2_TPM) by the contributors were used in our study. Cell annotations were provided by contributors. A total of 11 LUAD and 11 distant normal lung samples were included for further analyses. The expression levels of genes included in the proposed risk score were evaluated in different cell types.

Risk scores were calculated at the cell level and averaged for each LUAD sample. Then, 11 LUAD samples were divided into high- and low-risk groups according to the median risk score. Intercellular communication was modeled by using the CellPhoneDB Python package (version 2.1.7) (Efremova et al., 2020), and the significantly differentiated between the two groups ligand–target links were summarized by using the iTALK R package (version 0.1.0) (Wang Y. et al., 2019). Specifically, CellPhoneDB integrates existing datasets of cellular communication and new manually reviewed information, including the subunit architecture for both ligands and receptors. The normalized gene expression data and the cell annotations were analyzed by the relevant Python package, with subsampling, 50 rounds of iterations and 4 calculating threads. The iTALK R package is another useful toolkit for characterizing and visualizing intercellular communication. Growth factor, cytokine, checkpoint, and other types of intercellular communication were assessed by it. The top 20 ligand–target links with the greatest differences between the high- and the low-risk groups were visualized.

## Results

### Identification of Prognosis-Related ICAGs

Seven datasets downloaded from GEO were preprocessed as previously described, and a total of 1042 samples were eventually used as the discovery set. The results of eliminating batch effect were presented (see Supplementary Figure 1). Besides, 594 samples were downloaded from TCGA and used as the validation set, with 535 of them being LUAD samples and 59 of them being normal samples. The basic information of these datasets was summarized in Table 1. Besides, the characteristics of research subjects in each GEO dataset and the TCGA dataset were presented in Supplementary File 2. A total of 426 ICAGs were collected, and 354 genes, common in all datasets, were assessed in this study. Sixty-seven genes were significantly associated with the overall survival by univariate COX regression (see Supplementary File 4). Eight genes were finally selected because they presented non-zero coefficients in the fitted LASSO COX regression models, as it is shown in Table 2. The risk score was calculated as follows:

$$\begin{array}{cc} \text{RiskScore} & =0.0\text{9}0\text{22}\times \text{LAMB1}+0.0\text{9287}\times \text{GJC1}+0.\text{12437}\\ & \begin{array}{l} \times \text{CDH4}+0.\text{161}0\text{5}\times \text{GJB3}+0.\text{14827}\times \text{SPP1}\\ +0.\text{14339}\times \text{AFDN}+0.\text{16}0\text{16}\times \text{SKAP2}-0.\text{17981}\\ \times \text{ARHGEF6} \end{array} \end{array}$$

**TABLE 1:** Basic information of datasets included in this study.

Dataset ID	Number of samples	Brief introduction about the dataset	Number of deaths	Number of relapses
Discovery set	1042			
GSE19188	40	A genome-wide gene expression analysis on early-stage NSCLC	24	–
GSE30219	85	Identification of a group of metastatic-prone tumors in lung cancer according to “Off-context” gene expression defined by the authors	45	27 (83)
GSE31210	226	Gene expression analysis on pathological stage I–II lung adenocarcinomas	35	64 (226)
GSE31546	16	Development of an EGFR mutation gene expression signature to predict response and clinical outcome, and identification of genes associated with the EGFR-dependent phenotype	2	–
GSE37745	106	Biomarker discovery in NSCLC	77	–
GSE50081	127	Validation of a histology-independent prognostic gene signature for early-stage NSCLC, including stage IA patients	51	37 (124)
GSE68465	442	Gene expression-based survival prediction in LUAD	236	178 (178)
Validation set	535			
TCGA-LUAD	535	The LUAD cohort of TCGA, a landmark cancer genomics program, molecularly characterized over 20,000 primary cancer and matched normal samples spanning 33 cancer types.	187	–

**TABLE 2:** The prognostic ICAGs identified by using LASSO COX regression.

Gene	Coef	Encoded protein
ARHGEF6	−0.17981	Rho guanine nucleotide exchange factor 6
SKAP2	0.16016	Src kinase-associated phosphoprotein 2
AFDN	0.14339	Afadin, adherens junction formation factor
SPP1	0.14827	Secreted phosphoprotein 1
GJB3	0.16105	Gap junction beta-3 protein
CDH4	0.12437	Cadherin 4
GJC1	0.09287	Gap junction gamma-1 protein
LAMB1	0.09022	Laminin subunit beta-1

In addition, the nomogram we developed was presented in Supplementary Figure 2.

### Survival Analysis on the Risk Score

**FIGURE 2:**
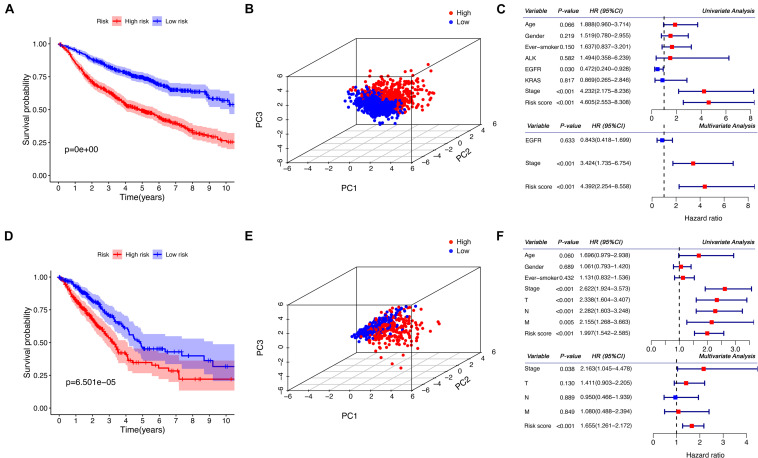
Prognostic analysis of the risk score in the discovery and the validation sets. **(A,D)** Kaplan–Meier curves for the comparison of the overall survival between the high- and the low-risk group. **(B,E)** The three-dimensional principal component analysis on the two groups. **(C,F)** Forest plots for the results of the univariate and multivariate COX regression analyses regarding the overall survival. **(A–C)** Show the results of the discovery set, while **(D–F)** show the results of the validation set. PC, principal component; HR, hazard ratio; CI, confidence interval.

Patients in the discovery and the validation sets were divided into high- and low-risk groups, according to whether their risk scores exceeded the median values. The Kaplan–Meier analysis with a log-rank test demonstrated that there were significant differences in the overall survival between the two risk groups. As shown in Figures 2A,D, the high-risk group has significantly worse overall survival compared to the low-risk group (*P* < 0.001). PCA also confirmed that patients in the two groups presented different patterns of gene expression and this finding is illustrated in Figures 2B,E. As seen, the blue points representing the low-risk group distributed together, while the red points representing the high-risk group in another part of space. This indicates significant differences in the gene expression levels between the two groups. Furthermore, as shown in Figures 2C,F, our risk score is significantly associated with the outcome in the univariate and the multivariate regression (*P* < 0.001), indicating the risk score was an independent predictive factor for the overall survival.

### Prediction of Relapse by Using Machine Learning

**FIGURE 3:**
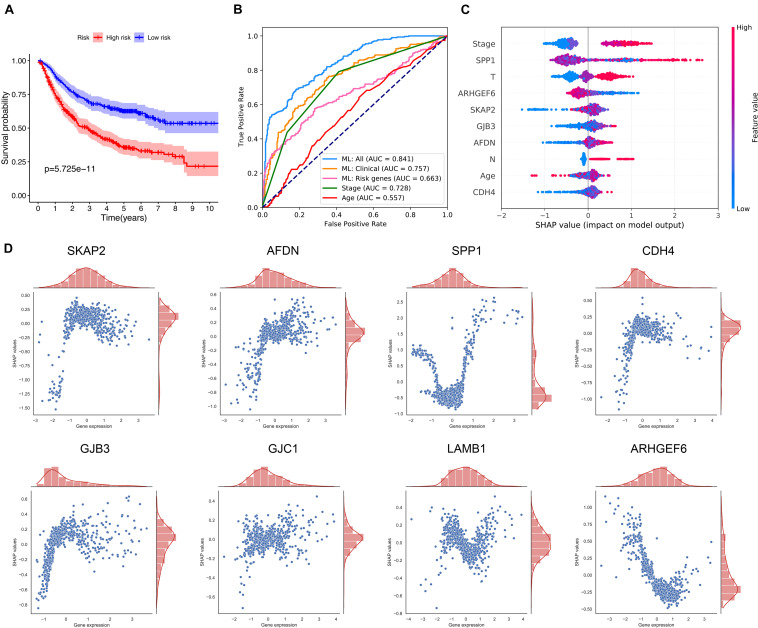
Prediction of LUAD relapse by using machine learning. **(A)** The Kaplan–Meier curve for the comparison of the disease-free survival between the high- and the low-risk groups. **(B)** The receiver operator characteristic curves for the explored predictive models or methods. **(C)** SHAP values show the variable impacts on the prediction results of the model using all clinical information and eight ICAGs. **(D)** The distributions of the SHAP values for each ICAG on the model using all clinical information and the eight ICAGs. ML, machine learning; AUC, area under curves; SHAP, shapley additive explanation.

Four datasets which provided relapse information were selected, including GSE68465, GSE30219, GSE50081, and GSE31210. The Kaplan–Meier analysis also proved that there were significant differences in the disease-free survival between the high- and the low-risk groups, as it is shown in Figure 3A.

The CatBoost algorithm was used to develop three machine-learning models based on different sets of variables to further elucidate the prognostic value of selected ICAGs. The first feature set includes only ICAGs selected by the LASSO COX regression and the model presented an AUROC of 0.663. The second includes only clinical information such as age, gender, and stage, and its AUROC was 0.757. The third set combined both clinical variables and the selected ICAGs, and a remarkable AUROC of 0.841 was achieved. The predictive performances of cancer stage and age were also assessed. The AUROCs of stage and age were 0.728 and 0.557, respectively. The machine-learning model, based on all features, outperformed all other predictive methods or models, as it is shown in Figure 3B.

SHapley Additive exPlanation values were assessed to evaluate the effects of each variable on the model’s output. SHAP values for the model using all features were shown in Figure 3C. Red color represents a high value of that feature, while blue color represents a low value. A positive SHAP value means that this feature value will increase the relapse risk, while a negative one represents a protective effect. The features in Figure 3C were ordered from top to bottom according to their importance, which was assessed by the average absolute SHAP values. Moreover, the relationships between the SHAP values and the gene expression levels were visualized in Figure 3D. SKAP2, AFDN, CDH4, GJB3, and GJC1 had similar positive correlation with SHAP values, while the expression level of ARHGEF6 is negatively correlated with SHAP values. The relationships between SHAP values and SPP1 or LAMB1 are not simply linear and needs more research.

### Gene Set Variant Analysis on the Validation Set

Gene Set Cancer Analysis was used for gene set variant analyses. Supplementary Figure 3 showed the analysis on SNVs of the eight ICAGs. Missense mutations were the most common variants, and the C > A and C > T SNVs were the most frequent variants. The median of variants per sample was 1. LAMB1, ARHGEF6, and CDH4 are the top mutated genes.

Analysis of CNVs was summarized in Supplementary Figure 4. From this figure, it is observed that LAMB1, GJC1, CDH4, and SKAP2 had frequent heterozygous amplification, while AFDN, SPP1, and GJB3 had frequent heterozygous deletion. Homozygous variants were less frequently observed in these genes, but CDH4, SKAP2, LAMB1, and AFDN had more frequent homozygous variants than other genes.

### Regulatory Networks and Methylation Modification

Micro RNA and lncRNA regulatory networks were visualized in Supplementary Figures 5A,B, respectively. The figures included key ICAGs, as well as the miRNAs and lncRNAs that target them. Methylation modification was summarized in Supplementary Figure 6. Five genes were shown in Supplementary Figure 6A, including CDH4, GJB3, LAMB1, SKAP2, and SPP1, of which expression levels were significantly correlated to their DNA methylation levels (*P*-value < 0.05). Besides, the expression levels of m6A regulatory genes were compared between the high- and the low-risk groups, as shown in Supplementary Figure 6B.

### Pathway Activity and Gene Set Enrichment Analyses

The results of pathway activity analysis were shown in Supplementary Figure 7. As shown in Supplementary Figure 7B, these genes were significantly correlated with the EMT activation. Besides, the eight genes also presented great effects on the inhibition of cell cycle. These results confirmed that the selected genes play an important role in cancer development and metastasis.

Expressions of 25,168 genes were compared between the high- and the low-risk groups in the validation set, and 10,830 genes were found to be differentially expressed with an adjusted *P*-value < 0.05. Gene set enrichment analyses were performed. DNA replication, chromosomal region, cell adhesion molecule binding and cadherin binding were significantly enriched GO terms, as shown in Supplementary Figures 8A,B. Cell cycle, focal adhesion, spliceosome, and homologous recombination were significantly enriched pathways according to the results of the KEGG pathway analysis (Supplementary Figures 8C,D). Among these DEGs, 991 had absolute fold changes greater than 2. Specifically, 727 genes were upregulated and 264 were downregulated in the high-risk group, compared to the low-risk group. The heatmap and volcano plot of these genes were plotted in Supplementary Figures 8E,F, respectively.

### Immunogenomic Landscape Analyses

**FIGURE 4:**
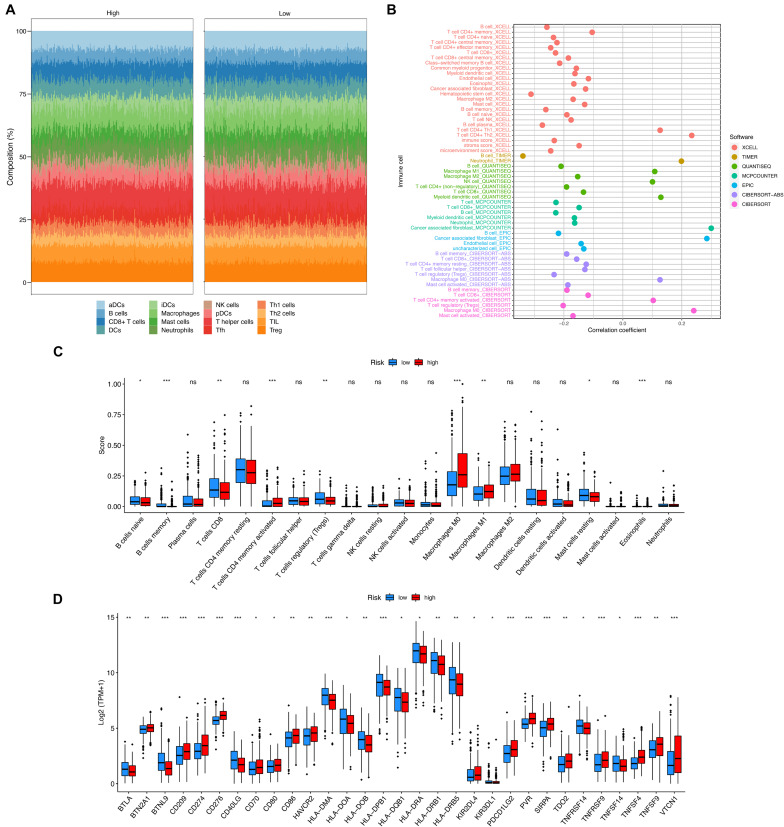
The immunogenomic landscape analyses on the validation set. **(A)** Compositions of different infiltrated immune cells estimated by ssGSEA. **(B)** Correlation between risk score and immune cells estimated by XCELL, TIMER, quanTIseq, MCP-counter, EPIC, and CIBERSORT. **(C)** Boxplot for the immune cell scores in the high- and the low-risk groups, estimated by CIBERSORTx. **(D)** Boxplot for the expression of an immune checkpoint gene in the high- and the low-risk groups. Adjusted P-values were showed as: ns, not significant; *P < 0.05; **P < 0.01; ***P < 0.001.

Various computational approaches regarding immune infiltration were conducted and summarized in Figure 4. As seen, most scores of immune cells were correlated with the risk score with a negative coefficient as shown in Figure 4B. This finding suggests that the high-risk group had fewer infiltrated immune cells. The scores provided by CIBERSORTx were compared between the two groups, as it is shown in Figure 4C. As seen, the high-risk group had fewer B cells naïve, B cells memory, T cells CD8, T cells regulatory, and Mast cells resting than the low-risk group. But more T cells CD4 memory activated, Macrophages M0, and Macrophages M1 were infiltrated in the high-risk group. More Eosinophils were infiltrated in the high-risk group, but the scores in both groups were too low such that the comparison of Eosinophils was not clear in the figure. Additionally, the expressions of 79 immune checkpoint genes were compared between the high- and the low-risk groups by using the two-sample Wilcoxon test. The checkpoint genes with a *P*-value < 0.05 were summarized in Figure 4D.

### Modeling of Intercellular Communication Based on a Single Cell RNA Sequencing Dataset

**FIGURE 5:**
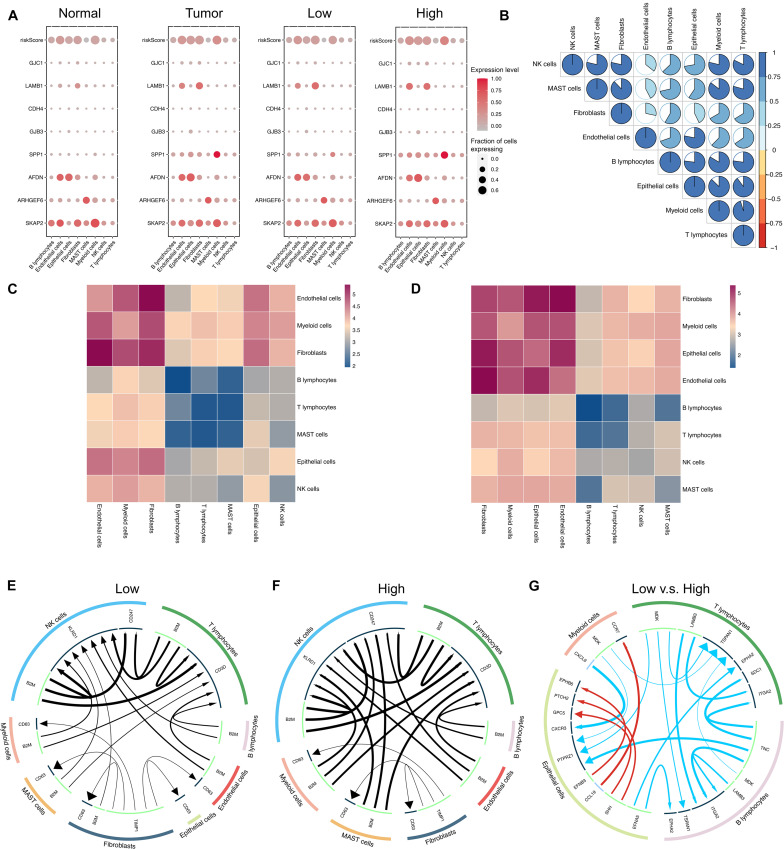
Modeling of intercellular communication based on a single cell RNA sequencing dataset. **(A)** Heatmap for the ICAG expression and the risk score in different groups. **(B)** Correlation of the risk scores in different cells. **(C,D)** Heatmaps for the intercellular communication generated by using CellphoneDB in the low-risk group **(C)** and the high-risk group **(D)**. **(E,F)** The main ligand–target expression between the different cells in the low-risk group **(E)** and the high-risk group **(F)**. **(G)** The top differences in the expression of ligand or target genes between the low- and the high-risk groups. **(E–G)** were generated by using the iTALK R package.

The Single-cell RNA sequencing dataset GSE131907 was used for further analysis in high resolution. A total of 11 LUAD and 11 distant normal lung samples were included in our study. In total, 42,679 normal cells and 45,149 tumor cells were assessed. A total of 22,977 and 22,172 tumor cells were assigned to high- and low-risk groups, respectively. The risk score and the expression levels of eight genes in different cells were assessed and compared, as demonstrated in Figure 5A. As seen, the expression patterns of these eight genes were different between the high- and the low-risk groups and also between the LUAD and normal groups. SPP1 was upregulated in all kinds of cells and especially the myeloid cells in the high-risk group compared to the low-risk group. AFDN and SKAP2 were upregulated in the epithelial cells of the high-risk group. These differences were also observed between tumor and normal samples. The correlation of risk scores between different cells was displayed in Figure 5B. Interestingly, the risk scores of various cells were positively correlated with each other.

Besides, intercellular communication was modeled by using CellphoneDB. The patterns of intercellular communication in the high- and low-risk groups were visualized in Figures 5C,D, respectively. As shown in Figures 5C,D, less communication was observed between B lymphocytes, T lymphocytes, NK cells, and MAST cells than between other cells, but in the high-risk group (shown in Figure 5D), there was more communication between epithelial cells and others in comparison with the low-risk group (shown in Figure 5C).

Furthermore, the differences in communication patterns between the two groups were compared by using the iTALK R package, and the results were displayed in Figures 5E–G. In Figure 5G, red color represents a gain of interaction, indicating upregulation of the ligand and the receptor genes, while blue color represents a loss of interaction, indicating downregulation of the ligand and the receptor genes. The thickness of edges indicates the expression level of the ligands, while the size of arrows indicates the expression level of receptors. Although some ligand–receptor links of autocrine were expressed more in epithelial cells, several ligand–receptor links were expressed significantly less in the high-risk group.

### Drug Sensitivity Analysis

The correlation of gene expression and drug sensitivity was assessed based on small molecules from CTRP and GDSC. The correlation coefficients and adjusted *P*-values were visualized in Supplementary Figure 9. It is easily observed that these eight genes were significantly correlated to the sensitivity of various drugs. Specifically, the positive correlations were commonly observed on AFDN, GJB3, LAMB1, and SPP1, while ARHGEF6 had negative correlations to most drugs.

## Discussion

In this study, we explored the prognostic potential of ICAGs in patients with LUAD. An eight-ICAG signature was eventually identified, and a risk score was accordingly derived based on the analysis of publicly available datasets. Machine-learning models were developed to predict tumor recurrency based on clinical information and the expression levels of selected ICAGs. Comprehensive analyses were conducted, including gene set variant, regulatory network, pathway activity, gene set enrichment, immunogenomic landscape, drug sensitivity analyses as well as modeling of intercellular communication based on single-cell RNA sequencing.

A large number of studies have shown that cell-to-cell communication participates in the construction of the TME of lung cancer, promotes the formation of lung cancer blood vessels, and accelerates tumor invasion and metastasis. Exosomes, cytokines, etc., can be released by tumor cells into the TME and blood circulation to promote tumor progression. For example, MALAT1 derived from exosomes has been found to be highly expressed in the serum of patients with non-small cell lung cancer, which can accelerate tumor migration and promote its growth (Gutschner et al., 2013). Another study showed that the absorption of vesicles of lung cancer cells by macrophages promotes the production of M2-like phenotype by tumor-associated macrophages, which in turn produces IL-1β, which is beneficial to the survival of tumor cells (Wang et al., 2011). In short, cell-to-cell communication can regulate the progression, metastasis, invasion, and proliferation of lung cancer through a variety of ways.

In this study, eight genes involved in intercellular communication were identified and prioritized in the present study. Previous research has reported the role some of them play in the progression of cancer. It was found that miR-135a can inhibit cancer stem cell-driven medulloblastoma development by directly repressing the expression of ARHGEF6 (Hemmesi et al., 2015) and ARHGEF6 might be a hub gene in colorectal cancer (Wang and Zheng, 2014). A high level of Afadin, which is the protein encoded by AFDN gene, was found to be associated with poor survival in breast cancer patients (Tabaries et al., 2019). GJB3, a member of the connexin gene family, was found to be a potential circulating biomarker for metastatic pancreatic cancer and might have a unique effect on cell death (Tattersall et al., 2009; Easton et al., 2019). Choi et al. (2020) detected the expression levels of GJC1 (Cx45) in HeLa cells and identified GJC1 as a major component of gap junctions. However, few studies focused on the role of these genes in LUAD. The findings in our study will contribute to a deeper understanding of the effects of these genes on the progression and relapse of LUAD.

The effects of other selected genes on LUAD or NSCLC have been previously reported by several studies. It was found that SPP1 may contribute to immune escape (Zhang et al., 2017), metastatic progression (Chiou et al., 2019), and second-generation epidermal growth factor receptor tyrosine kinase inhibitor resistance (Wang X. et al., 2019). Tanaka et al. (2016) revealed that SKAP2 is related to tumor-associated macrophage infiltration and facilitates the metastatic progression of lung cancer in mise. The prognostic value of SKAP2 was also reported in previous studies using bioinformatics analyses (Kuranami et al., 2015; Tanaka et al., 2016; Chen et al., 2019). Studies showed that CDH4 could be regulated by miR-211-5p to inhibit the proliferation, migration, and invasion of LUAD (Zhang et al., 2020). Besides, the aberrant expression of ligand–receptor pair LAMB1-ITGB1 was identified within tumor cells in LUAD (Chen et al., 2020). In our study, the selected ICAGs except ARHGEF6 presented a positive coefficient, indicating that the upregulation of the expression levels results in poor prognosis. Our study confirms the results of these prior studies and may facilitate other research on the functions of these genes.

The prognostic value of these eight ICAGs was evaluated by survival analysis. Significant differences were observed in the overall survival between the high- and the low-risk groups. The risk score derived by LASSO COX regression was proved to be an independent predictive factor for the overall survival in LUAD. Moreover, a machine-learning model based on clinical information and expression of the eight ICAGs accurately predicts LUAD recurrence better than other predictive methods or models. Our risk score and model could contribute to the determination of the severity of LUAD and to stratify patients’ prognosis.

Further comparisons between the high- and the low-risk groups were performed, including gene set enrichment and immune infiltration analyses. GO and KEGG pathway analyses demonstrated that cell cycle, focal adhesion, DNA replication, and cell adhesion molecule binding were enriched by DEGs between the high- and the low-risk groups. Besides, it was shown by immune infiltration analyses that there were significant differences in the TME between the two groups. Previous studies have already reported that misleading communication within and between tumor cells and immune cells contributes to immune escape, metastatic progression, and drug resistance of LUAD (Tanaka et al., 2016; Chen et al., 2020). In addition, as shown in Supplementary Figure 7, these eight genes are also involved in cell growth cycle pathways such as cell cycle pathway and DNA damage response pathway. Sex hormone receptor pathways are also related to these eight genes, such as hormone AR pathway and hormone ER pathway. In addition, PI3K/AKT, RTK, RAS/MAPK, TSC/mTOR pathways can also interact to promote the occurrence and development of lung cancer. In our study, these enriched pathways and microenvironment differences were likely to result from aberrant changes in the intercellular communication. More research and experiments are required to shed light on the effects of aberrant intercellular communication in cancers.

A single-cell RNA sequencing dataset was used to further model the intercellular communication in LUAD. The ICAG expressions in different cells were assessed in the present study, evaluating the communication relationships between different cells and comparing the differences in the communication patterns between the high- and the low-risk groups. Changes in transferring information may be the key mechanism in immune escape and therapy resistance of LUAD. Our study provides insight into the potentially therapy target role of the ICAGs in LUAD.

According to drug sensitivity analysis, we found that these eight genes are related to AS605240 (PI3K inhibitor), AZD8055 (mTOR inhibitor), AZD-7762 (cell cycle checkpoint kinase), vinblastine (a lung cancer targeted drug), and other drugs. The results are consistent with the results of our previous analysis. This further supports the results of the previous pathway analysis and the conclusion of the article.

Several limitations of this study should be considered. Firstly, the analyses of this study were conducted based on public datasets, without verification or validation from *in vitro* or *in vivo* biochemical experiments. Thus, the revealed eight-ICAG signature and our machine-learning models require further validation in large-scale prospective studies to demonstrate their robustness. Secondly, various approaches to estimating immune infiltrated cells or modeling intercellular communication were used in the present study, but their results were not entirely consistent. However, any one of these computational approaches is not a “Gold Standard.” In contrast, they provide different perspectives to estimate what we are interested in. That is exactly the reason why we tried as many approaches as possible in the study, instead of drawing our conclusion based on anyone of them. Lastly, other factors, such as circular RNAs and proteins, involved in intercellular communication, were not included in our study. Multi-omics data may facilitate a deeper understanding of the pathogenesis and optimize the prediction of survival in LUAD.

## Conclusion

In this study, we comprehensively assessed the role of ICAGs in LUAD, identifying eight key ICAGs with prognostic value and developing a risk score as well as machine learning models to predict the prognosis for patients with LUAD. These genes may contribute to understanding the pathological mechanism of LUAD, and could also be considered as potential therapeutic targets.

## Data Availability Statement

Publicly available datasets were analyzed in this study. This data can be found here: TCGA data were available on the project website at https://www.cancer.gov/about-nci/organization/ccg/research/structural-genomics/tcga, while the GEO datasets were available at https://www.ncbi.nlm.nih.gov/geo/.

## Ethics Statement

The study was an analysis of third-party anonymized publicly available datasets with pre-existing institutional review board (IRB) approvals.

## Author Contributions

Q-YZ and L-PL: conception, design, data analysis, and interpretation. RG and Y-WL: administrative support. Q-YZ: collection and assembly of data. All authors: writing and final approval of manuscript.

## Conflict of Interest

The authors declare that the research was conducted in the absence of any commercial or financial relationships that could be construed as a potential conflict of interest.

## Publisher’s Note

All claims expressed in this article are solely those of the authors and do not necessarily represent those of their affiliated organizations, or those of the publisher, the editors and the reviewers. Any product that may be evaluated in this article, or claim that may be made by its manufacturer, is not guaranteed or endorsed by the publisher.

## Footnotes

1
http://bioinfo.life.hust.edu.cn/GSCA/#/

2
http://cbioportal.org

## Supplementary Material

Supplementary File 1 Supplementary Figures 1–9.

Supplementary File 2 The baseline characteristics of research subjects in each GEO dataset and the TCGA dataset.

Supplementary File 3 List of intercellular communication-associated genes included in the study.

Supplementary File 4 The results of univariate COX regression regarding overall survival on the discovery set.

## Abbreviations

NSCLC: non-small-cell lung cancer
LUAD: lung adenocarcinoma
TME: tumor microenvironment
ICAG: intercellular communication-associated gene
DEG: differentially expressed gene
GEO: Gene Expression Omnibus
FPKM: fragments per kilobase million
TCGA: The Cancer Genome Atlas
TPM: transcripts per million
KEGG: Kyoto Encyclopedia of Genes and Genomes
GO: Gene Ontology
LASSO: the least absolute shrinkage and selection operator
PCA: principal component analysis
CatBoost: Categorical Boosting
AUROC: the areas under receiver operator characteristic curve
SHAP: SHapley Additive exPlanation
GSCA: Gene Set Cancer Analysis
SNV: single-nucleotide variant
CNV: copy number variation
miRNA: micro RNA
CTRP: the Cancer Therapeutics Response Portal
GDSC: the Genomics of Drug Sensitivity in Cancer
lncRNA: long non-coding RNA
GRCh38.p13: Genome Reference Consortium Human Build 38 patch release 13
m6A: N6-methyladenosine
ssGSEA: single-sample gene set enrichment analysis.
