# Supplementary material for: A Novel Intercellular Communication-Associated Gene Signature for Prognostic Prediction and Clinical Value in Patients With Lung Adenocarcinoma
Source: Front Genet. 2021 Aug 23;12:702424. doi: 10.3389/fgene.2021.702424 (PMC8419521; doi:10.3389/fgene.2021.702424)
Supplement: Supplementary File 1 — Supplementary Figures 1–9. [file Data_Sheet_1.pdf]

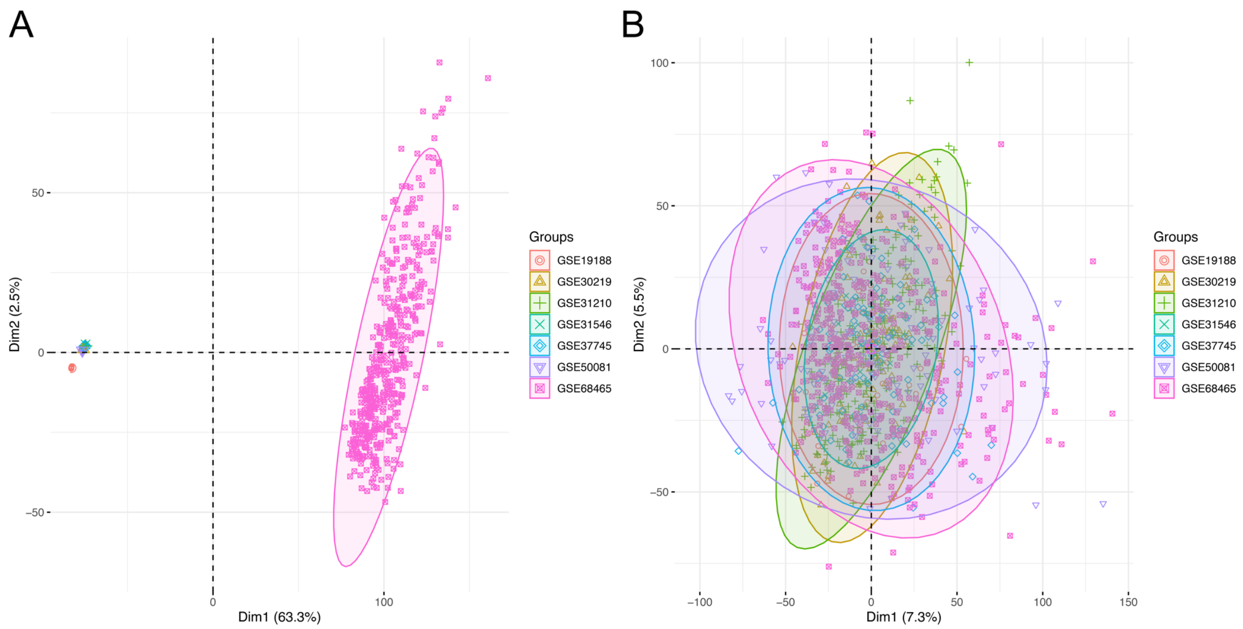

Figure S1. Principal component analysis of GEO datasets before and after eliminating batch effect. Principal component analysis was used to present the results of eliminating batch effect. (A) and (B) are the analysis results of GEO datasets before and after eliminating batch effect, respectively. As seen, the batch effect was observed in a clear way in (A), while in (B), the samples of different datasets distributed in several concentric circles. This figure proved that the batch effect was significantly eliminated in our research.

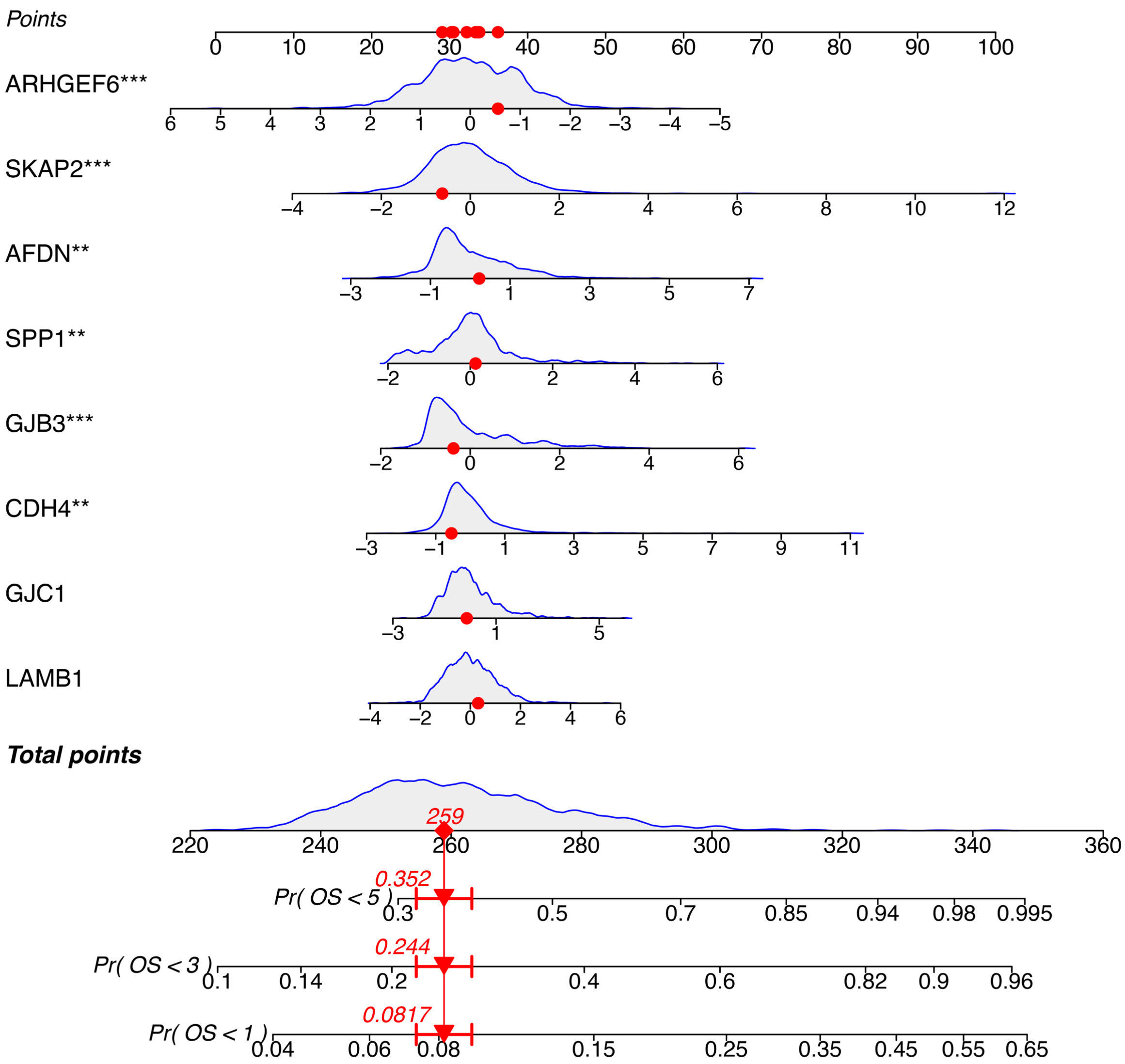

Figure S2. A nomogram using prognosis-related ICAGs to predict overall survival. The nomogram was developed by using the functions provided in the R package regplot (version 1.1). The red parts represent the prediction results of one sample from the GEO datasets.

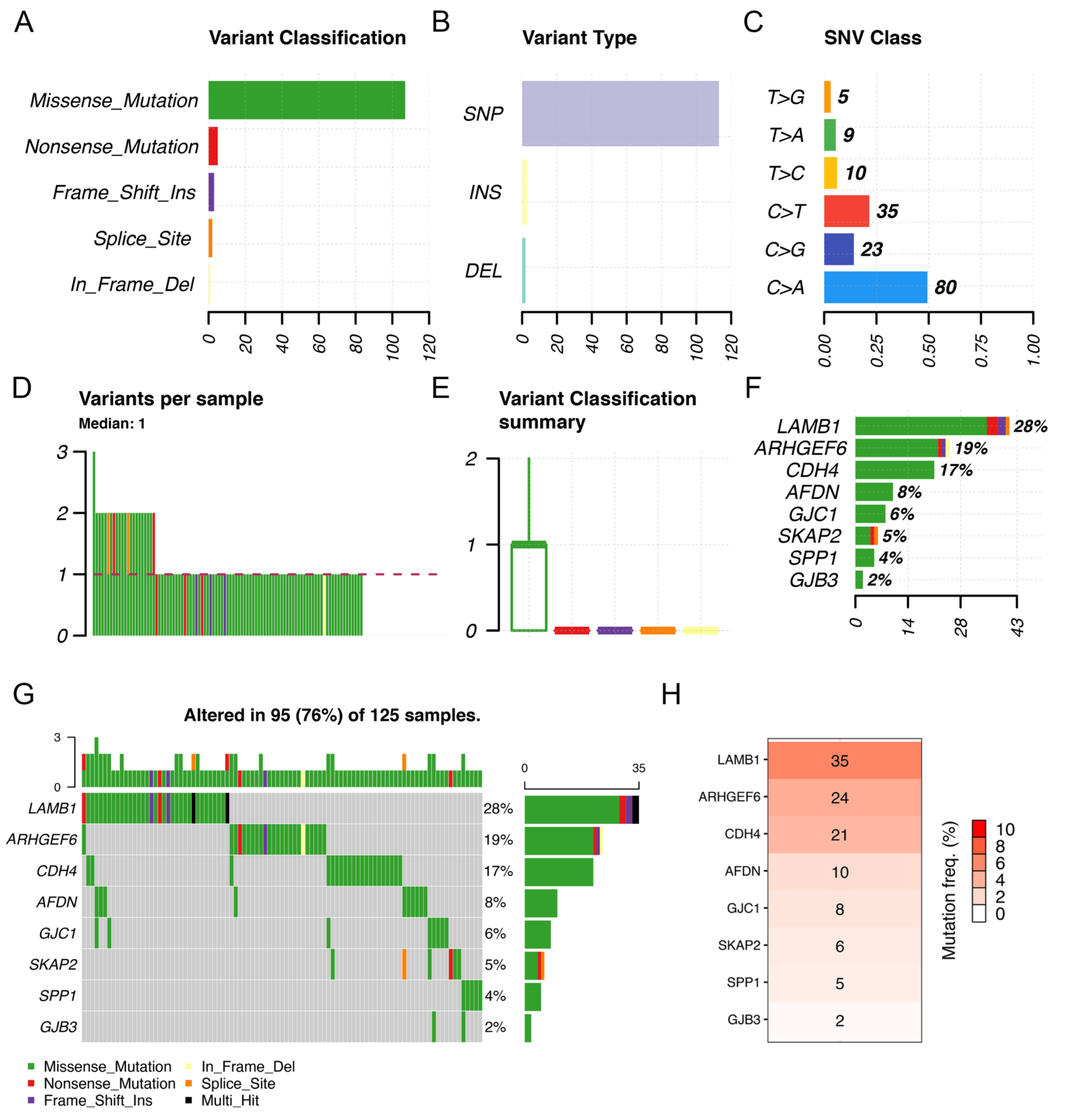

Figure S3. Analyses on SNV of the 8 selected ICAGs on the validation set. **(A)** Variant classification; **(B)** Variant type; **(C)** SNV class counts; **(D)** Variants per sample; **(E)** Variant classification summary; **(F)** Variant ratio; **(G)** Waterfall plot for SNV; and **(H)** Mutation frequency of each gene.

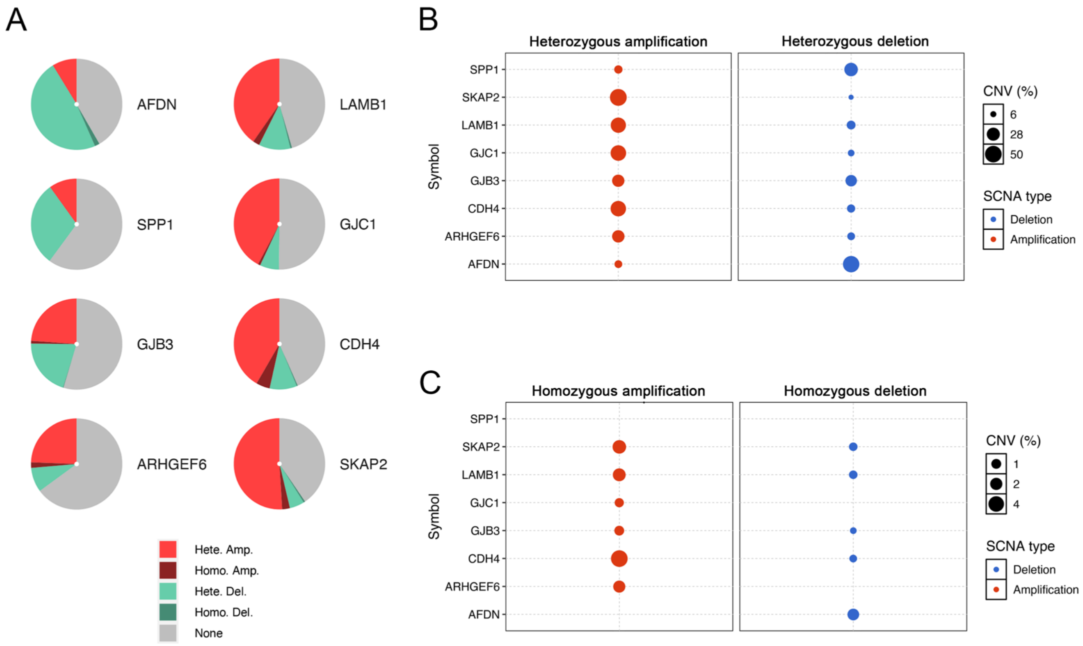

Figure S4. Analyses on CNV of the 8 selected ICAGs on the validation set. **(A)** CNV summary for each ICAG; **(B)** Heterozygous amplification and deletion of each ICAG. **(C)** Homozygous amplification and deletion of each ICAG.

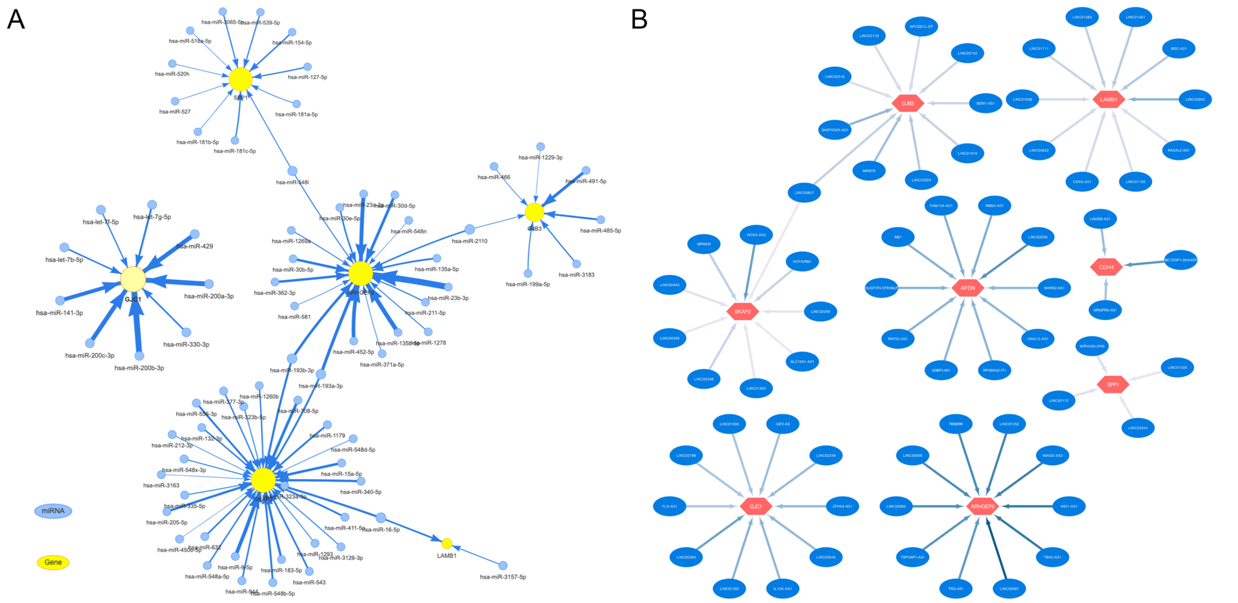

Figure S5. Regulatory network analyses on the 8 selected ICAGs based on all TCGA data. **(A)** The miRNA-mediated regulatory network. A node represents a miRNA or a gene, while an edge represents a regulatory relationship between a miRNA and a gene. Node size represents the node's degree, and edge width is positively correlated to the absolute value of the correlation coefficient. **(B)** The lncRNA regulatory network. A blue circle represents a lncRNA while a red polygon represents a gene. Deeper color of edge represents a stronger correlation.

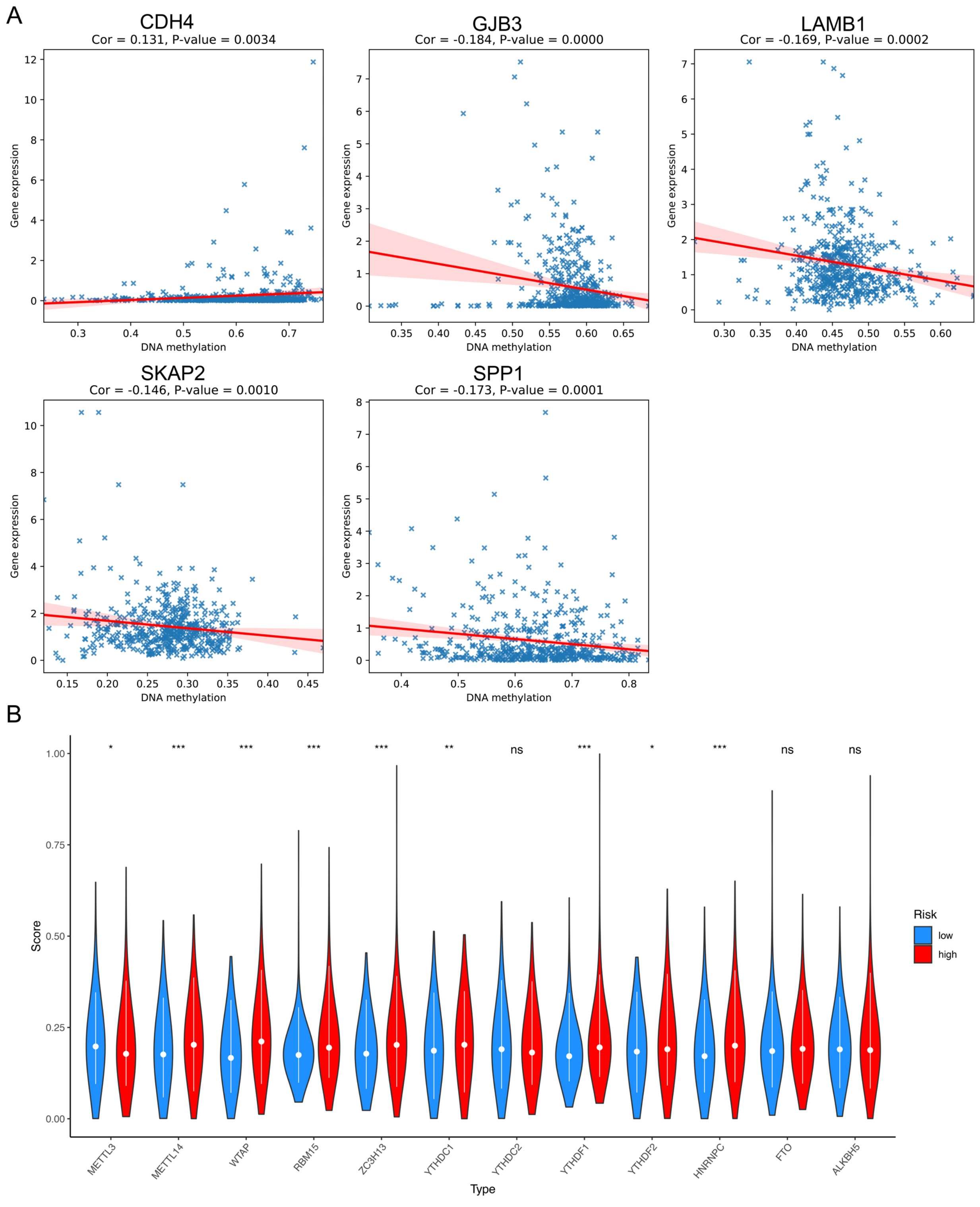

Figure S6. DNA methylation and m6A analyses on the validation set. **(A)** The scatterplots show the relationships between expression and DNA methylation levels of ICAGs. Each red line represents a fitted linear regression model, and relevant light-red zone represents the confidence interval **(B)** The expression levels of m6A regulatory genes were compared between high- and low-risk groups.

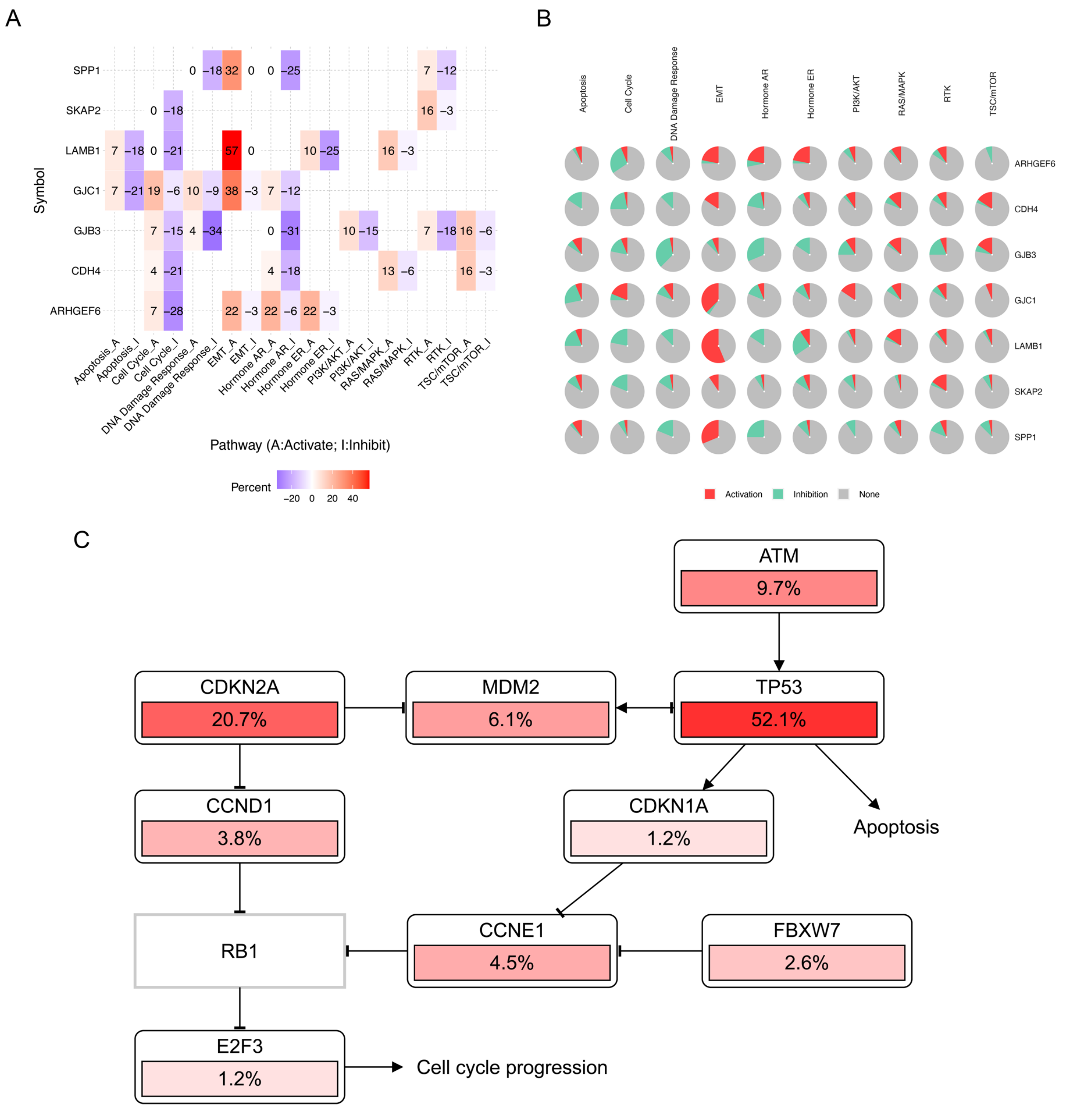

Figure S7. Pathway activity analyses on the 8 selected ICAGs. Heatmap **(A)** and pie charts **(B)** for the percentage of cancers in which the gene has an effect on the pathway among the 32 cancer types of TCGA by using GSCA (percentage = number of activating or inhibiting cancer types / 32 * 100%). **(C)** A pathway was significantly affected by the selected ICAGs, which was obtained by using the cBioPortal online platform.

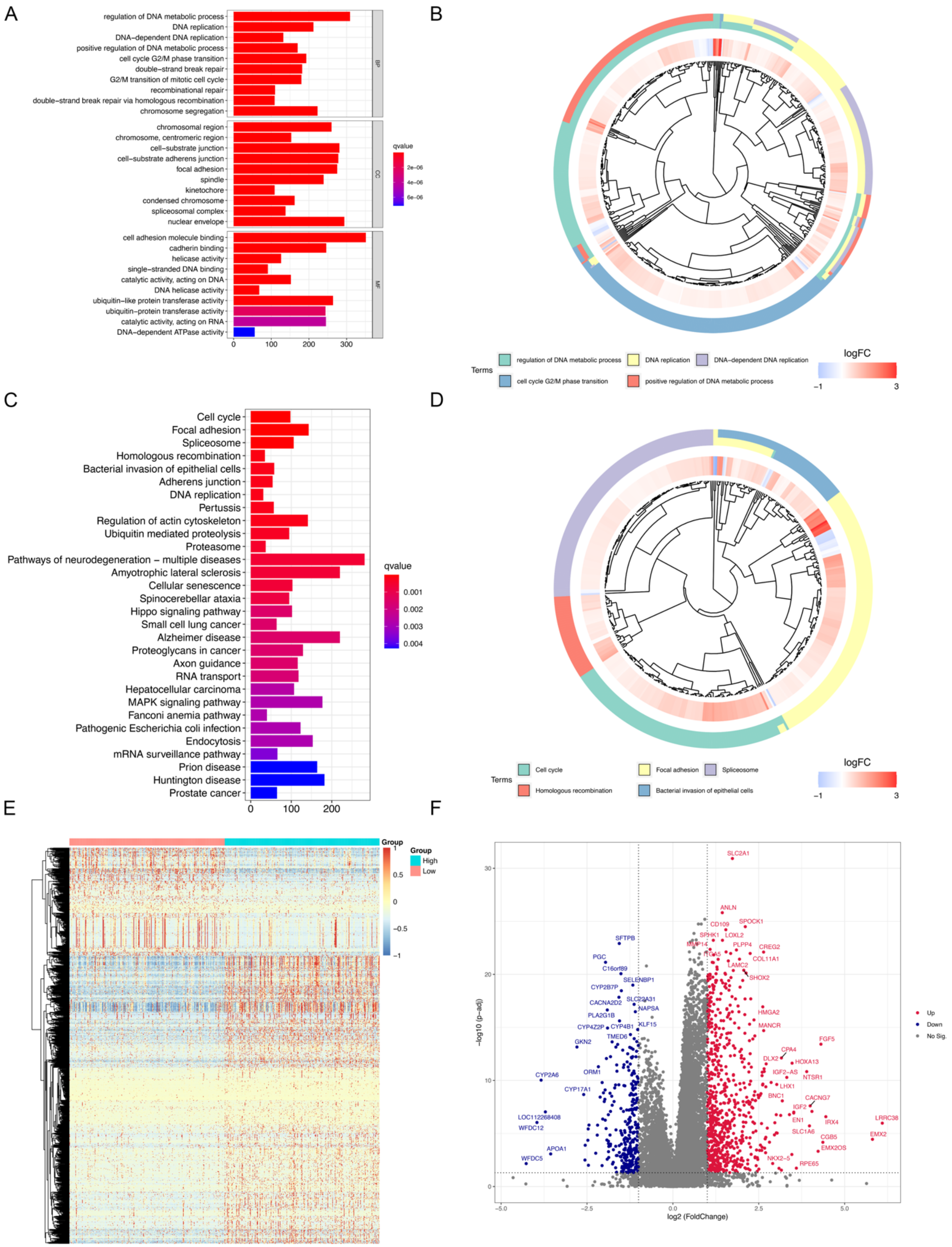

Figure S8. Gene set enrichment analyses on DEGs between the high- and the low-risk groups of the validation set. (**A**) and (**B**) show the bar plot and cluster plot of significant GO functional

items, respectively. (**C**) and (**D**) show the bar plot and cluster plot of significant KEGG pathways, respectively. **(E)** Heatmap of DEG expressions. **(F)** Volcano plot of DEGs.

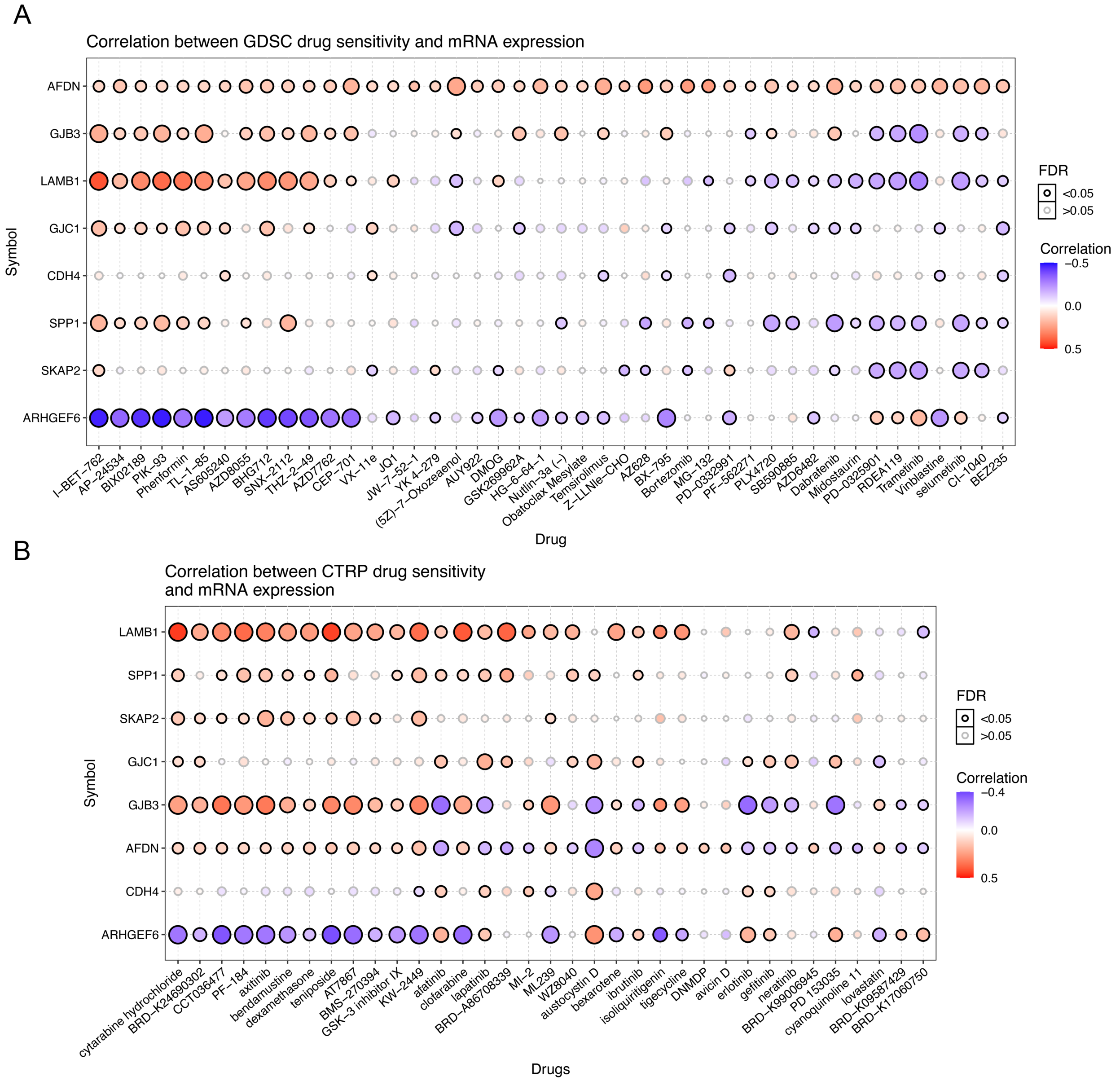

Figure S9. Drug sensitivity analysis based on the GDSC **(A)** and the CTRP **(B)** research. The Spearman correlation between ICAG expression levels and drug sensitivity was calculated.
